# Green Synthesis of NiO-SnO_2_ Nanocomposite and Effect of Calcination Temperature on Its Physicochemical Properties: Impact on the Photocatalytic Degradation of Methyl Orange

**DOI:** 10.3390/molecules27238420

**Published:** 2022-12-01

**Authors:** Sirajul Haq, Anum Sarfraz, Farid Menaa, Nadia Shahzad, Salah Ud Din, Hanadi A. Almukhlifi, Sohad A. Alshareef, Ethar M. Al Essa, Muhammad Imran Shahzad

**Affiliations:** 1 Department of Chemistry, University of Azad Jammu and Kashmir, Muzaffarabad 13100, Pakistan; 2 Departments of Public Health and Environmental Engineering, California Innovations Corp., San Diego, CA 92037, USA; 3 US-Pakistan Centre for Advanced Studies in Energy, National University of Science and Technology (NUST), Islamabad 44000, Pakistan; 4 Department of Chemistry, Faculty of Science, University of Tabuk, Tabuk 71491, Saudi Arabia; 5 Department of Civil Engineering, Isra University, Amman 11622, Jordan; 6 Nanosciences and Technology Department (NS & TD), National Center for Physics (NCP), Islamabad 44000, Pakistan

**Keywords:** nickel stannate, calcination, photodegradation, methyl orange, mineralization, sustainability

## Abstract

*Background:* Nickel stannate nanocomposites could be useful for removing organic and toxic water pollutants, such as methyl orange (MO). *Aim:* The synthesis of a nickel oxide–tin oxide nanocomposite (NiO-SnO_2_ NC) via a facile and economically viable approach using a leaf extract from *Ficus elastica* for the photocatalytic degradation of MO. *Methods:* The phase composition, crystallinity, and purity were examined by X-ray diffraction (XRD). The particles’ morphology was studied using scanning electron microscopy (SEM). The elemental analysis and colored mapping were carried out via energy dispersive X-ray (EDX). The functional groups were identified by Fourier transform infrared spectroscopy (FTIR). UV–visible diffuse reflectance spectroscopy (UV–vis DRS) was used to study the optical properties such as the absorption edges and energy band gap, an important feature of semiconductors to determine photocatalytic applications. The photocatalytic activity of the NiO-SnO_2_ NC was evaluated by monitoring the degradation of MO in aqueous solution under irradiation with full light spectrum. The effects of calcination temperature, pH, initial MO concentration, and catalyst dose were all assessed to understand and optimize the physicochemical and photocatalytic properties of NiO-SnO_2_ NC. *Results:* NiO-SnO_2_ NC was successfully synthesized via a biological route using *F. elastica* leaf extract. XRD showed rhombohedral NiO and tetragonal SnO_2_ nanostructures and the amorphous nature of NiO-SnO_2_ NC. Its degree of crystallinity, crystallite size, and stability increased with increased calcination temperature. SEM depicted significant morphological changes with elevating calcination temperatures, which are attributed to the phase conversion from amorphous to crystalline. The elemental analysis and colored mapping show the formation of highly pure NiO-SnO_2_ NC. FTIR revealed a decrease in OH, and the ratio of oxygen vacancies at the surface of the NC can be explained by a loss of its hydrophilicity at increased temperatures. All the NC samples displayed significant absorption in the visible region, and a blue shift is seen and the energy band gap decreases when increasing the calcination temperatures due to the dehydration and formation of compacted large particles. NiO-SnO_2_ NC degrades MO, and the photocatalytic performance decreased with increasing calcination temperature due to an increase in the crystallite size of the NC. The optimal conditions for the efficient NC-mediated photocatalysis of MO are 100 °C, 20 mg catalyst, 50 ppm MO, and pH 6. *Conclusions:* The auspicious performance of the NiO-SnO_2_ NCs may open a new avenue for the development of semiconducting p–n heterojunction catalysts as promising structures for removing undesirable organic pollutants from the environment.

## 1. Introduction

For the scientific community, environmental protection has recently become one of the greatest worries due to several industries that are severely affecting the environment and the fast rise of organic contaminants used for different industrial purposes [1]. Water pollution is a serious issue among several other environmental problems that are rooted in the unchecked discharge of industrial influents containing toxic organic dyes and heavy metals [2,3]. Organic dyes are known to cause allergies, dermatitis, genetic mutations, cancers, vomiting, and cardio-respiratory disorders in humans [4,5,6].

Methyl orange (MO) is an anionic azo dye that is used in the paper, pharmaceuticals, food, cosmetics, and textile industries [7,8,9]. Owing to its slow biodegradability, MO remains in the environment for a long period and represents an organic pollutant [7,8,9]. Several methods such as adsorption, incineration, membrane filtration, coagulation, and absorption have been used for dye removal [9], but they did not prove efficient as they generated disposal problems, came at a high cost, and were time-consuming. Compared to the other alternatives, semiconductor-based photocatalytic degradation has been proven to be an efficient, rapid, economical, and environmentally friendly process [10,11,12]. The photocatalysis process obeys the basic principle of the advanced oxidation process (AOP) to generate reactive radical species with UV radiation to catalyze organic pollutant degradation [7,13,14].

Different types of metal and metal oxide nanoparticles have been synthesized for various applications, such as catalysis, energy storage devices, dye-based solar cells, and biomedicines [2,7,13,14,15,16,17,18,19,20,21,22,23].

NiO is a p-type semiconductor with a wide band gap (3.6–4.0 eV) and exclusive optical and electrochemical properties that display high conductivity [24,25]. NiO is widely applied in numerous fields such as photocatalytic agents, gas sensors, adsorbents, and fuel cells [26]. NiO NPs have been prepared by several methods including spin coating, pulsed laser deposition, sol–gel methods, and green methods [27].

Tin oxide nanoparticles (SnO_2_ NPs) are n-type semiconductors with a wide band gap (3.6 eV) [28]. Due to their strong oxidizing power, different morphologies, non-toxic nature, and high photochemical stability, SnO_2_ NPs are one of the best choices for photocatalysts [19,21,22]. Owing to their properties such as low cost, electro-catalytic activity, high conductivity, and high chemical stability, SnO_2_ NPs have also been used extensively in the arena of electrochemical sensing [29]. SnO_2_ NPs have been fabricated using physical and chemical methods such as laser ablation, the sol–gel method, spray pyrolysis, microemulsion, microwaves, electrochemical deposition, co-precipitation, and hydrothermal, sonochemical, solvothermal, and non-aqueous routes [20,30,31]. Importantly, the paucity of reports on the photodegradation of dyes with NiO NPs and SnO_2_ NPs compared to TiO_2_ NPs and ZnO NPs [7,13,14,18,21,22,32] justifies the present study.

The reaction medium, capping agents, and reducing agents are the three main decisive factors in the synthesis and stabilization of nanomaterials [33]. In this study, NiO-SnO_2_ NCs have been biosynthesized using a leaf extract of *F. elastica* for the purification of wastewater contaminated with MO. *F. elastica*, commonly known as the rubber tree, is an important medicinal plant belonging to the Moraceae family [34]. The green synthetic route is a more facile method of synthesizing nanomaterials. The method is user friendly, easy to operate, and low-cost, requires no elevated reaction conditions (mean high pressure and temperature), and presents no solvent and separation issues. The final product can be easily separated via a simple centrifugation/filtration process. The proper mechanism of the green process is not known yet, but it is assumed that the hydroxyl group containing biomolecules acts as a reducing and capping agent. The crystallographic properties, optical properties, and structural and surface functional groups of the synthesized NiO-SnO_2_ NCs were studied using XRD, DRS, SEM, and FTIR, respectively. The NiO-SnO_2_ NCs were used as photocatalysts for the photodegradation of MO under different initial dye concentrations, catalyst doses, and pHs. The effect of the calcination temperature on the physicochemical and photocatalytic properties of NiO-SnO_2_ NCs was also examined.

## 2. Results and Discussion

### 2.1. XRD Analysis

**Figure 1:**
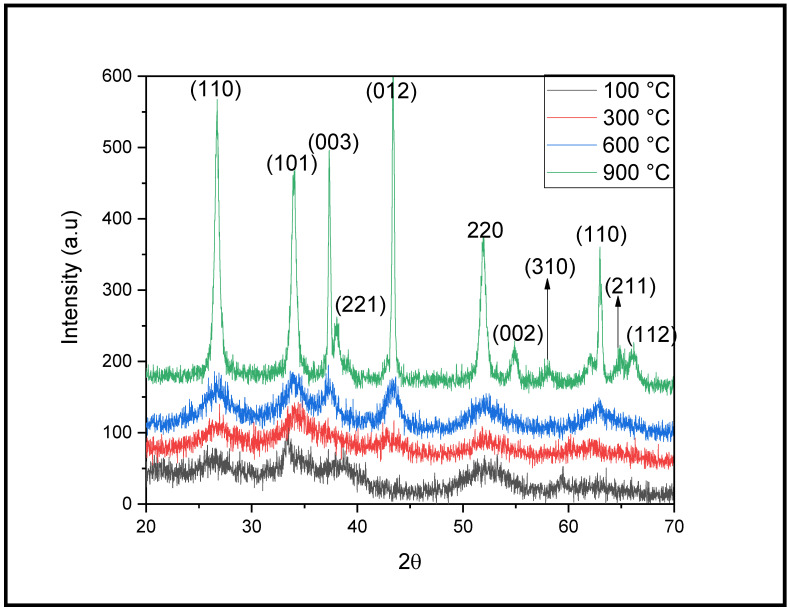
XRD diffractograms of NiO-SnO_2_ NCs calcined at the indicated temperature.

The purity, composition, and structure of the NiO-SnO_2_ NCs were investigated by XRD. The XRD patterns of NiO-SnO_2_ NCs calcined at 100, 300, 600, and 900 °C are shown in Figure 1. The XRD patterns of the NiO-SnO_2_ NCs calcined at 100 °C display no diffraction peaks, showing that the sample is amorphous. The diffraction bands for the samples calcined at 300, 600, and 900 °C appeared at 2*θ* positions 37.32, 43.43, and 62.94, corresponding to Miller indices (003), (012), and (110), respectively. All the XRD bands are well matched with the Bragg reflections listed in the reference JCPDS card #00-022-1189, which reveals the rhombohedral geometrical shape of calcined NiO. The diffraction bands that appeared at the 2*θ* position are 26.276, 34.22, 38.01, 52.02, 54.83, 58.00, 64.77, and 66.13, corresponding to Miller indices (110), (101), (221,) (220), (002), (310), (112), and (112) in the XRD pattern of NiO-SnO_2_ NC calcined at 300, 600, and 900 °C, revealing the tetragonal geometry of nano-sized SnO_2_ corresponding to the JCPDS card #00-001-0657. The intensity of the diffraction bands increases with increasing calcination temperature from 300 to 900 °C. This demonstrates that the degree of crystallinity and crystallite size gradually increases with calcination temperature. The crystallite size (with lattice strain in parentheses) calculated by the Debye–Scherrer equation considering the full width at half maximum (FWHM) of the diffraction bands are 23.42 (1.88%), 29.83 (1.36%), 41.59 (0.98%), and 78.21 (0.17%) nm for the NiO-SnO_2_ NC calcined at 100, 300, 600, and 900 °C, respectively. The increase in crystallite size and decrease in lattice strain strongly suggest the high stability of NiO-SnO_2_ NC at a high calcination temperature.

### 2.2. SEM Analysis

**Figure 2:**
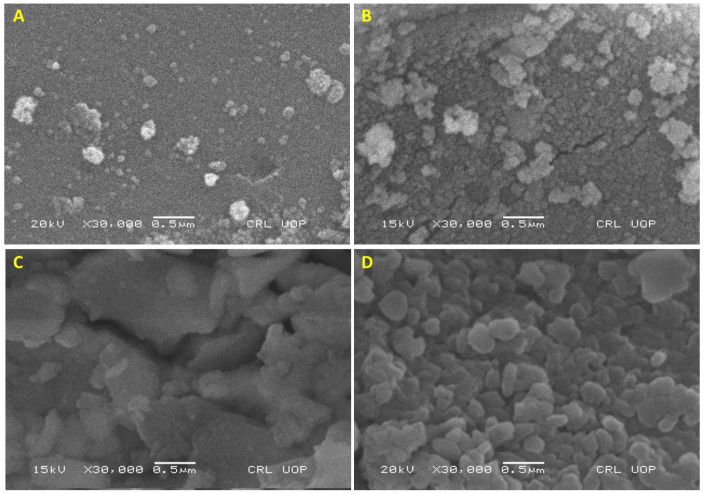
SEM micrographs of NiO-SnO_2_ NCs calcined at (**A**) 100 °C, (**B**) 300 °C, (**C**) 600 °C, and (**D**) 900 °C. The magnification and scale bar are indicated.

SEM analysis was performed to explore the effect of calcination temperature on the morphology of NiO-SnO_2_ NCs. SEM micrographs (at high magnification) of SnO_2_ NPs calcined at 100, 300, 600, and 900 °C reveal significant morphological changes with elevating calcination temperatures (Figure 2A–D). The sample dried at 100 °C seemed to be a fine powder, in which several aggregates could be noticed (Figure 2A). Besides these few aggregates, the powder is smoothly distributed, and no boundary is visible. The increase in the calcination temperature up to 300 °C led to the formation of a cluster with a grainy surface, and they can be differentiated from each other due to their visible boundary. Along the grain formation, the aggregates seen in image A (Figure 2A) also increased in size, leading to the formation of a compact solid structure, and few narrow cracks appeared in the sample (Figure 2B). The formation of a compact solid became evident in Figure 2C. Indeed, upon increasing the calcination temperature up to 600 °C, the cracks widened and the breaking of the large compact structure led to the formation of several small structures. Further, an increase in the calcination temperature up to 900 °C promoted the formation of small particles of various sizes and shapes (Figure 2D). The particles formed are different from each other as they have well-defined boundaries. Moreover, some larger plate surface particles seem to be made up of three or more particles. The high surface energy might be responsible for the fusion of small particles, which led to the formation of some larger structures. These changes, which occur when increasing the temperature, could be attributed to the phase conversion from amorphous to crystalline, nucleation, growth, fragmentation, or the rearrangement of particles.

### 2.3. UV–Vis DRS Analysis

**Figure 3:**
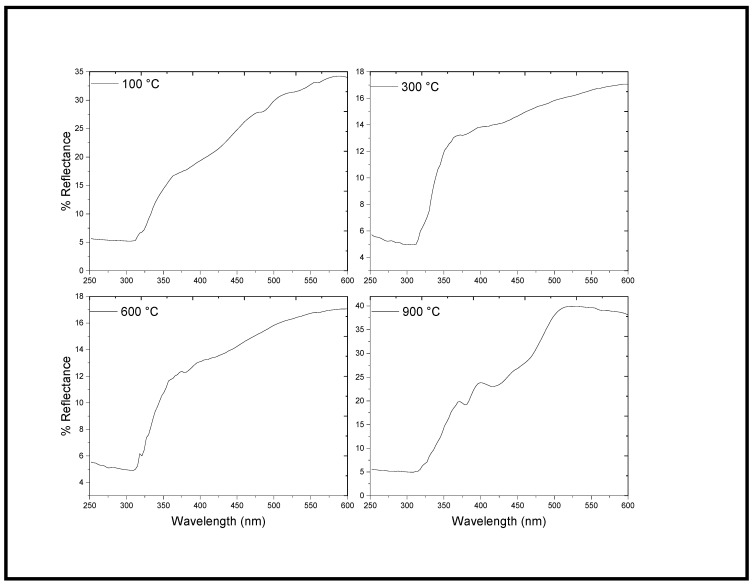
DRS spectra of the NiO-SnO_2_ NCs calcined at the indicated temperature.

The energy gap is an important feature of semiconductors and determines their applications in optoelectronics. To calculate the energy band gap of NiO-SnO_2_ NCs, DRS was utilized. The DRS spectra of the NiO-SnO_2_ NC calcined at 100, 300, 600, and 900 °C are shown Figure 3. Then, the sharp rising portion in the UV–vis curve was aligned with the x-axis of the DRS spectrum to calculate the wavelength of the transmittance edge [21,35].

**Figure 4:**
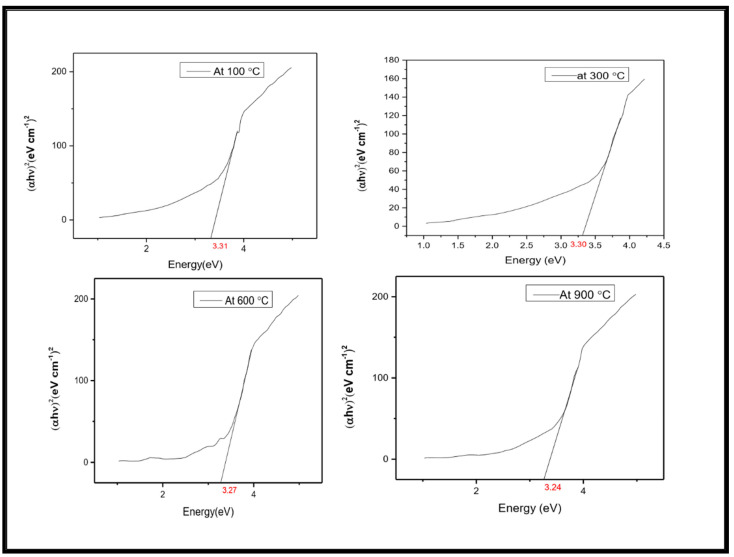
Determination by Tauc’s plot of the band gap of NiO-SnO_2_ NCs calcined at the indicated temperature.

The transmittance edge obtained for NiO-SnO_2_ NCs calcined at 100, 300, 600, and 900 °C were 324, 325, 326, and 345 nm, respectively. All the samples show significant absorption in the visible region and a blue shift was seen with increasing calcination temperatures. The band gap energy of NiO-SnO_2_ NC calcined at 100, 300, 600, and 900 °C, determined through Tauc plot, were 3.31, 3.30, 3.27, and 3.24 eV, respectively (Figure 4). With an increase in the calcination temperature, the energy band gap decreases, which might be due to dehydration and the formation of compacted large particles [36].

### 2.4. FTIR Analysis

**Figure 5:**
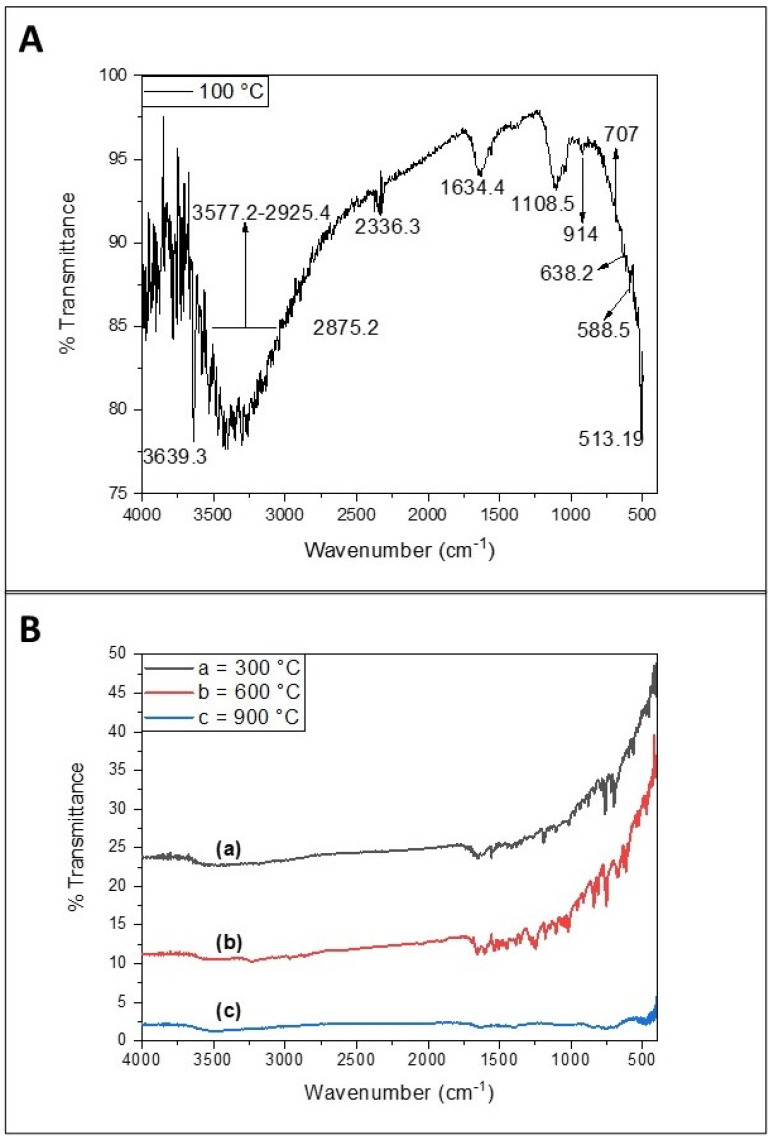
The FTIR spectrum of NiO-SnO_2_ NCs calcined at (**A**) 100 °C and (**B**) over 100 °C (temperature indicated).

The FTIR spectrum of the biosynthesized NiO-SnO_2_ NCs calcined at 100 °C (Figure 5A) displays a broad peak ranging from 3577.2 to 2925.4 cm^−1^, with one peak at 1634.4 cm^−1^ that may be associated with the bending bond and stretching bond of adsorbed water molecules, respectively. The strong band at 3639.3 cm^−1^ is assigned to the vibration of the hydroxyl group, whereas the peak at 2875.02 cm^−1^ is attributed to CH stretching [37]. The band at 914 cm^−1^ is assigned to Sn-O-Ni stretching [38]. The band at 707 cm^−1^ is characteristic of metal-oxygen-metal bridging. The Sn-O band was observed at around 588.5 cm^−1^ and the Sn-O-Sn band was observed at around 638.2 cm^−1^ [39].

The FTIR spectra of NiO-SnO_2_ NCs calcined over 100 °C (at 300, 600, and 900 °C) exhibit the same transmittance peaks as discussed in the case of NiO-SnO_2_ NCs calcined at 100 °C (Figure 5B). However, the reduction in intensity or complete absence of some peaks is observed with increasing calcination temperatures. Additionally, the intensity of the peaks is reduced in the FTIR spectrum of the NiO-SnO_2_ NC sample annealed at 900 °C. These data show that the calcined clusters are no longer likely to be nanoclusters. Indeed, the calcination temperature alters the structural properties along with the chemical composition. The bio-based synthesized NPs possess many biomolecules on the surface, and upon increasing the calcination these biomolecules are decomposed. Similarly, NPs synthesized in aqueous media are always in hydroxide (OH) form, and when they are calcined, the condensation reaction causes the elimination of water molecules, leaving the metal oxide. Such a phenomenon has been explained by the loss of its hydrophilic character, which consequently leads to a decrease in the surface OH and the ratio of oxygen vacancies at a high temperature [40,41,42]. Thus, changes in the FTIR bands are attributed to chemical alterations that occur due to the calcination process at higher temperatures.

### 2.5. EDX Analysis

**Figure 6:**
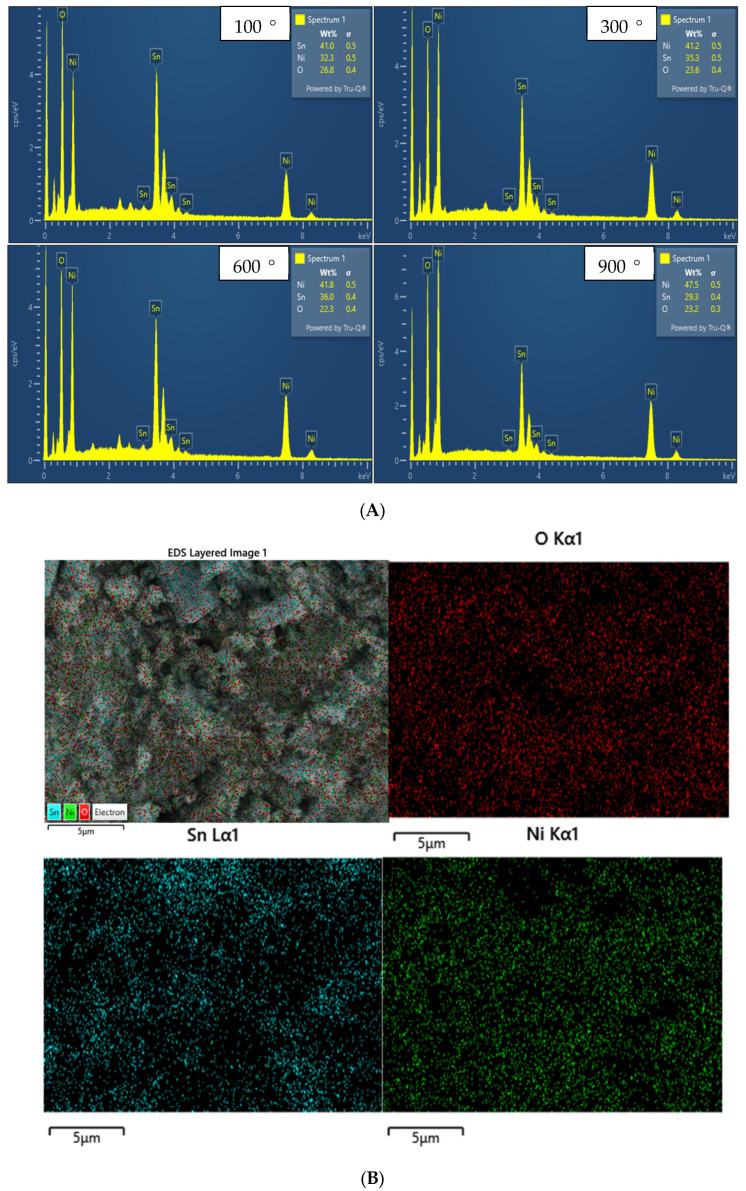
(**A**) EDX spectra of NiO-SnO_2_ NCs calcined at different temperatures. (**B**) EDX mapping images of NiO-SnO_2_ NCs calcined at 900 °C; red = O; blue = Sn; green = Ni.

The bands present in the EDX spectra shown in Figure 6A, attributed to Ni, Sn, and O, confirmed the synthesis of highly pure NiO-SnO_2_ NCs. The sharp intense band at 0.45 keV is due to the O, and the deceased intensity with calcination temperature might be due to the removal of water molecules. The bands at 0.8, 7.5, and 8.25 keV are assigned to Ni, and the intensity of these peaks increases with increasing calcination temperature. The signals that appear in the range of 3 to 4.35 keV are ascribed to Sn, and the intensity is seen to decrease with increasing calcination temperature. The random increase or decrease in the weight percent might be due to the composition of the area selected for EDX analysis. It might also be possible that the increase in the weight percent of Ni and the decrease in the weight percent of Sn are due to the rapid crystal growth of NiO as compared to SnO_2_ with increasing calcination temperature. The weight percent of the oxygen is reduced to a greater extent, which might be due to the condensation of metal hydroxide to metal oxide with the evaporation of water molecules at a higher calcination temperature. This is also evident from the FTIR spectra, where the intensity of the bands assigned to O-H vibration is reduced with increasing calcination temperature.

The presence of the Ni, Sn, and O in the sample was also confirmed through the color mapping of the NiO-SnO_2_ calcined at 900 °C, which is shown in Figure 6B. The red dots denote O, the blue dots represent Sn, and the green dots correspond to Ni. As can be seen from Figure 6B, that the maximum surface exposure is exhibited by Ni, followed by Sn, while O exhibits minimal surface exposure. The photographic results are in correspondence with the EDX results, and the larger weight percent of Ni in the sample is due to its high surface exposure.

### 2.6. Photocatalytic Activity

**Figure 7:**
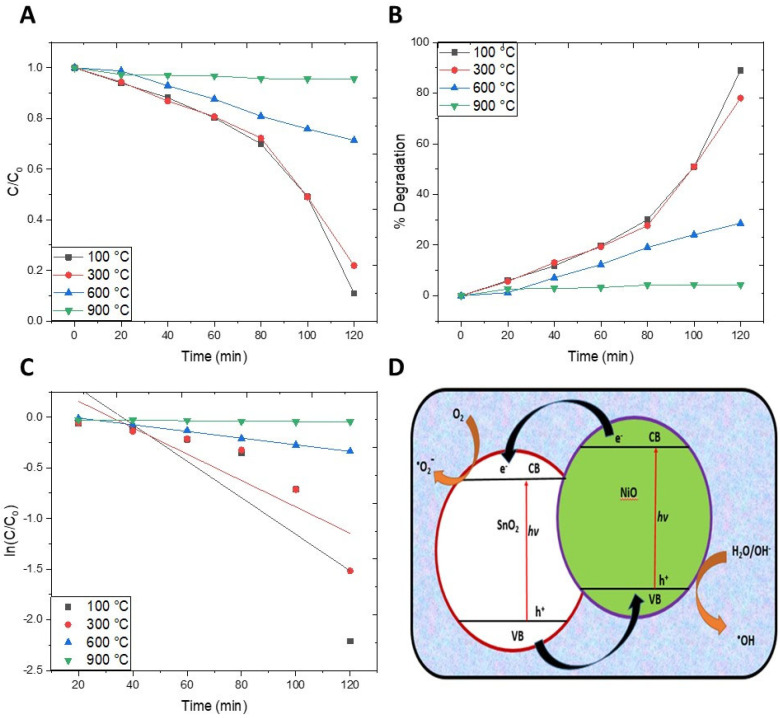
(**A**) Degradation profile, (**B**) percentage degradation, (**C**) kinetic isotherms/rate constant, and (**D**) schematic mechanism of photodegradation of MO in the presence of NiO-SnO_2_ NCs calcined at the indicated temperature.

The photocatalytic performance of NiO-SnO_2_ NC toward MO was investigated across the entire light spectrum (Figure 7). A total of 50 ppm aqueous (deionized water) solution of MO was prepared, and 50 mL from this solution was moved to the reaction tank/vessel, along with 20 mg of the catalyst (0.4 g/L). To attain an adsorption–desorption equilibrium, this reaction mixture was stirred for 30 min in the dark. The reaction mixture was monitored by a UV–vis spectrophotometer where the λ-max of the MO was revealed to be 464 nm. At different time points (i.e., 20, 40, 60, 80, 100, and 120 min), the color liquid samples calcined at 100 °C were examined spectrophotometrically, and a decrease in λ-max was noted as a function of time, showing a gradual decrease in the chromophore responsible for the light absorbance at 464 nm. The same experiment was repeated for the mineralization of MO with NiO-SnO_2_ NC calcined at 300, 600, and 900 °C, for which similar observations were made. No shift was noted at these high temperatures, but a gradual reduction in the λ-max of the MO could be observed as a function of both increasing time and calcination temperature.

The degradation parameters were calculated by applying mathematical equations (Figure 7A–C), and a model of photocatalytic degradation is proposed (Figure 7D).

The *C*/*C_o_* vs. time plots reveal a successive decrease in the absorbance with increasing time duration, strongly suggesting the degradation of MO molecules (Figure 7A). It was seen that the light absorbance capacity of the solution was higher with increasing calcination temperature, which demonstrates that the photocatalytic efficacy of NiO-SnO_2_ NCs decreases with an increase in temperature.

The percentage photocatalytic degradation of MO determined by Equation (1) (where *C_o_* is the initial concentration and *Ce* is the concentration of dye after time (*t*)), shows that 89.04, 78.07, 28.06, and 4.38% of the MO was degraded by NiO-SnO_2_ NC calcined at 100, 300, 600, and 900 °C, respectively (Figure 7B). The highest degradation was obtained for NiO-SnO_2_ NC calcined at 100 °C, and sequential degradation (%) decreased with increased calcination temperature.

(1) $$\%\;degradation=(C_{o}-Ce/C_{o})\;\times 100$$

The kinetic study of the photocatalytic reaction was carried out by applying the pseudo-first-order kinetic model (Equation (2)), and a straight line was obtained when *ln*(*C*/*C_o_*) was plotted against time (*t*) (Figure 7C), suggesting that the reaction follows pseudo-first-order kinetics [15,21,43]. The degradation rate constant (*k*) was found to be 0.019, 0.014, 0.004, and 0.0002 min^−1^ for the photocatalytic reaction carried out in the presence of NiO-SnO_2_ NC NPs calcined at 100, 300, 600, and 900 °C, respectively (Figure 7C).

*In* (^*C*^/*C_o_*) = −*kt* (2)

A schematic diagram of the proposed photocatalytic reaction mechanism (Figure 7D) shows a transfer of charges across the p–n junction (NiO-SnO_2_). The photocatalytic reaction mechanism depends on the band gap energy of the catalyst and light energy or wavelength. Semiconductors are mostly used as catalysts as they sensitize the irradiations of redox process (light simulated) due to their electronic structure, which is characterized by a vacant conduction band and a filled valence band [15]. When a photon of equal or greater energy than the band gap energy of the semiconductor hits its surface, the valence band electrons are perturbed and are shifted towards the conduction band [13,14,15]. The donor molecules are oxidized by the holes left in the valence band, which then react with the water molecules to generate hydroxyl radicals. The strong oxidizing ability of these radicals brings about the mineralization of MO. The conduction band electrons produce superoxide ions upon reaction with oxygen species, thereby inducing redox reactions. These electrons and holes produce the necessary products after sequential oxidation and reduction reactions with the specie adsorbed on the semiconductor surface [44].

Electron excitation occurred from the valence band (VB) to the conduction band (CB) for both oxides, leaving a positive hole in the valence band upon the exposure of the NiO-SnO_2_ NCs to light.

The transformation holes occurred from the valence band of SnO_2_ to the valence band of NiO. Consequently, excited electrons shifted from the conduction band of NiO to the conduction band of SnO_2_ [22]. This transformation of charge carrier entity to either side results in the complete elimination of the possible recombination of positive and negative charges. The excited electrons react with the dissolved oxygen, leading to the generation of oxygen anions. These react with water, resulting in the formation of hydroxyl radicals and hydrogen peroxide. On the other hand, the positive holes react with water to form hydroxyl radicals, and the radicals produced by both processes lead to the photodegradation of MO. The overall mechanism is summarized in the chemical reactions (Equations (3)–(9)) given below, which correspond with those reported in the literature [22].

(3) $$\left(NiO/SnO_{2})heterojunction\overset{hv}{\to }NiO(h_{VB}^{+})+SnO_{2}(e_{CB}^{-}\right)$$

(4) $$SnO_{2}(e_{CB}^{-})+O_{2}\to SnO_{2}+O_{2}^{∙-}$$

(5) $$O_{2}^{∙-}+H_{2}O\to HO_{2}^{∙}+OH^{-}$$

(6) $$HO_{2}^{∙}+H_{2}O\to HO^{∙}+H_{2}O_{2}$$

(7) $$H_{2}O_{2}\to 2OH*$$

(8) $$NiO(h_{VB}^{+})+OH^{-}\to NiO+OH^{∙}$$

(9) MO+OH∗→degradation of methyl orange

### 2.7. Factors Affecting the Photocatalytic Activity

**Figure 8:**
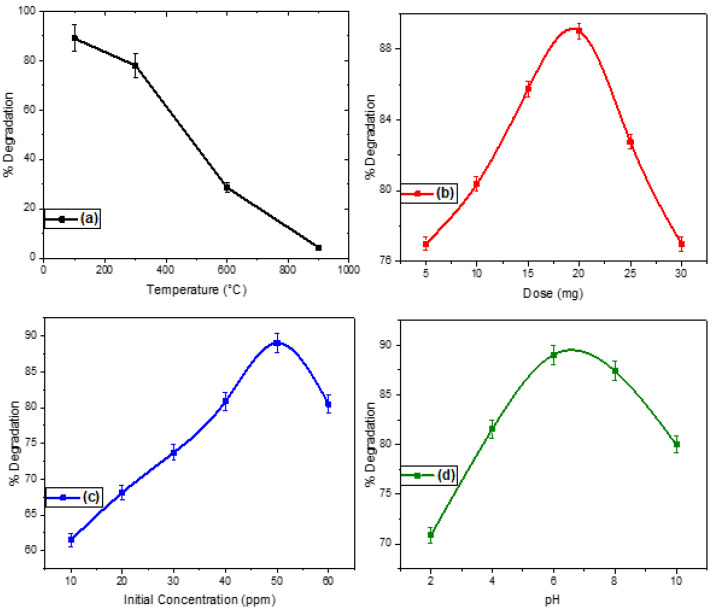
Effect of (**a**) annealing temperature (100–900 °C), (**b**) pH (2–10), (**c**) catalyst dose (5–30 mg), and (**d**) initial methyl orange (MO) concentration (10–60 ppm) on the NiO-SnO_2_ NC-induced photodegradation of MO. For each effect to be analyzed, all other variables were optimized and kept constant.

Photocatalytic efficiency is considerably affected by the initial concentration, pH, catalyst dose, and annealing temperature (Figure 8a–d). The photocatalytic activity of the NiO-SnO_2_ NCs calcined 100 °C was found to be higher than that of the other samples. Thus, the photocatalytic efficacy of this (most efficient) catalyst was evaluated at different initial MO concentrations, catalyst doses, and pHs. The same experiments were repeated with catalysts calcined at higher annealing temperatures.

#### 2.7.1. Effect of the Annealing Temperature

In the present study, the NiO-SnO_2_ NCs were calcined at 100, 300, 600, and 900 °C, and the samples calcined at 100 °C elicited the highest photocatalytic activity as compared to the analog catalysts calcined at higher temperatures (Figure 8a). This enhanced photocatalytic behavior might be due to the small particles size, which was confirmed by both XRD and SEM. In the literature, it has been reported that the surface area is decreased with an increase in particle size. With a lower surface area, a smaller number of binding sites are available for the photocatalytic reaction [13,14,45]. This may explain the decrease in the photocatalytic degradation of MO in the presence of NiO-SnO_2_ NCs calcined at higher concentrations.

#### 2.7.2. Effect of the Catalyst Dose

The photocatalytic degradation of MO was investigated using various quantities (i.e., 5, 10, 15 20, 25, and 30 mg) of NiO-SnO_2_ NCs (Figure 8b). The results reveal that the degradation of MO was increased with an increase in the catalyst dose up to 20 mg, whereas further increases led to a gradual decrease in the degradation of the MO. The higher MO degradation observed with up to 20 mg of catalyst can be explained by the increased number of active sites, while more than 20 mg of catalyst likely retarded the MO degradation by blocking the penetration of light.

#### 2.7.3. Effect of Initial Concentration of MO

Various concentrations of MO (i.e., 10, 20, 30, 40, 50, and 60 ppm) were exposed to NiO-SnO_2_ NC to check the effect of the initial concentration on the degradation process (Figure 8c). A progressive increase in the percent degradation was seen when increasing the initial concentration of MO up to 50 ppm, whereas a further increase in the concentration led to a gradual decline in photocatalytic activity. This effect up to 50 ppm may be due by the maximum light penetration and effective interaction between the catalyst and the light enhancing the degradation process. However, beyond 50 ppm, the blockage of light radiation by the dye molecules decreases the photocatalytic activity.

#### 2.7.4. Effect of pH

The solution of MO was treated at different pH levels (i.e., 2, 4, 6, 8, and 10) to investigate the effect of pH on the photocatalytic efficacy of the NiO-SnO_2_ NCs while all other parameters such as catalyst dose (50 ppm), type of catalyst (NiO-SnO_2_ NC calcined at 100 °C), and dose of the catalyst (20 mg) were kept constant throughout the procedure (Figure 8d). The results show that the maximum degradation occurred at pH 6, demonstrating that pH also significantly affects the photocatalytic degradation of MO. This may be because an acidic medium promotes degradation by elevating photo-oxidation as compared to an alkaline medium. The photo-oxidation is accelerated due to the free radicals produced during the reaction of the organic compound with the dissolved oxygen under sunlight. The particle surface charge is greatly affected by the variation in pH, which determines particle agglomeration. The suppression of Van der Waals’ forces around pH 6 probably enhances the dispersion of NiO-SnO_2_ NCs, which ultimately increases the degradation of MO [46].

## 3. Materials and Methods

### 3.1. Reagents

Analytical grade chemicals, including nickel sulphate hexahydrate, tin (II) chloride dehydrate, sodium hydroxide, and methyl orange, were purchased from Sigma Aldrich (St. Louis, MO, USA). They were used without any further purification. All the working solutions were prepared using deionized water.

### 3.2. Plant

The leaves of *F. elastica* were amassed from Chahlla bandi (Latitude 34°23′35″ N; Longitude 73°28′2″ E), Muzaffarabad district, Azad Kashmir region, Pakistan (Figure S1, Supplementary Material). The taxonomical identification of the plant was confirmed (under voucher #FE22PB) by Dr. Hamayun Shaheen, an Associate Professor and experienced herbalist at the Department of Botany, University of Azad Jammu and Kashmir, Muzaffarabad, Pakistan.

### 3.3. Preparation of the Plant Extract

The amassed leaves of *F. elastica* were thoroughly washed using deionized water to expunge the dust particles and were subsequently shade dried. A total of 50 g of the dried leaves was introduced to 1000 mL of boiled deionized water in an airtight jar and aged for 5 h. The crude extract was filtrated and centrifuged at a speed of 4000 rpm to draw out suspended impurities. The upper layer was stored at 4 °C for further experiments.

### 3.4. Green Synthesis of NiO-SnO_2_ NC

The preparation of the nanocomposite required three main steps. In the first step, 2.24 g of nickel sulphate hexahydrate was dissolved in 50 mL of distilled water in a titration flask, and 20 mL of the prepared extract was added. The reaction mixture was stirred and heated to 60 °C for 30 min, and sodium hydroxide solution was added to adjust the pH to 10. The prepared Ni(OH)_2_ gel was aged for 6 h. In the second step, following an adjusted protocol previously published [15,19], 0.98 g of tin (II) chloride dehydrates were dissolved in 50 mL of distilled water, and 20 mL of the plant extract was added. The sodium hydroxide solution was added to adjust the pH to 10. The reaction mixture was stirred and heated to 60 °C for 30 min, and the prepared Sn(OH)_2_ was aged for 6 h. In the third step, for the synthesis of NiO-SnO_2_ NC, the aged NiO(OH)_2_ and Sn(OH)_2_ gels were mixed with vigorous stirring in a beaker and heated to 50 °C for 4 h. The resulting gel was aged for 12 h and filtered to collect a solid product. The final product was washed thrice with deionized water and dried in an oven at 100 °C, followed by a calcination process using various temperatures (300, 600, and 900 °C) for 3 h in a muffle furnace. Using a pestle and a mortar, the final products were crushed into a fine powder and stored in polyethylene bottles.

### 3.5. Physicochemical Characterizations

#### 3.5.1. XRD, SEM, EDX, UV–Vis, and FTIR

XRD is a non-destructive method widely used to assess the crystallinity and structure of solid samples [13,14,15,16]. The fluctuations in the crystallographic properties with calcination temperature were analyzed using an XRD Panalytical X-pert Pro (PANanalytical B.V., EA Almelo, The Netherlands) that was operated at 2-theta (20–80°). The Debye–Scherrer equation was applied while considering the width at the half maxima to articulate the crystallite sizes.

SEM is a high-resolution surface imaging method in which the incident electron beam scans across the sample surface and interacts with the sample to generate backscattered and secondary electrons that are used to create an image of the sample surface microarchitecture [13,14,15,16]. The morphology of the prepared SnO_2_ NPs was analyzed via SEM model JSM 5910 (JEOL, Tokyo, Japan). Oxford Ultim^®^ Max EDS detector (Oxford instruments, Abingdon, UK) coupled with SEM was used for elemental analysis, percentage purity, and colored mapping.

To study the optical behavior of the SnO_2_ NPs, the UV–Vis DRS model Lambda 950 (PerkinElmer, Waltham, MA, USA) was operated in the range of 400–1000 nm [15,47]. The band gap calculation was conducted from the obtained data using Tauc’s plot [15,21].

FTIR is an instrumental technique used to identify chemical bonds present in organic and inorganic compounds by measuring their absorption of infrared radiation over a range of wavelengths [13,14,15,16]. FTIR analysis was performed over the range of 4000–400 cm^−1^ using a Nicolet 6700 (ThermoFisher Instruments, Waltham, MA, USA) apparatus to recognize the functional groups present in the SnO_2_ NPs calcined at various temperatures (range of 100–900 °C). The shift in the λ-max of the MO and the alteration (decrease) in the absorbance intensity were recorded using a double beam UV–vis spectrophotometer model Unicam UV500 (Thermo Spectronic, Rochester, NY, USA) during photocatalytic degradation.

#### 3.5.2. Photocatalytic Assay

Using a solar light lamp (US-800 (250 W)) as a light source, the photocatalytic potency of the NiO-SnO_2_ NCs was checked over time against the MO (during the mineralization of MO) in aqueous solution. Each experiment was performed in a double-walled glass reactor using 50 mL of MO solution (50 ppm) and 20 mg of calcined NiO-SnO_2_ NCs (0.4 g/L). The reactor was enclosed in an aluminum foil. The adsorption–desorption equilibria were achieved by agitation for 30 min in the dark followed by illumination with an artificial solar light source. Then, 3 mL of the reaction (liquid) mixture was centrifuged for 5 min at 4000 rpm to remove any suspended particles. Then, the clear sample was analyzed by double beam UV–vis spectrophotometry (model Unicam UV500; Thermo Spectronic, Rochester, NY, USA), and the changes (decrease) in the absorbance maxima (λ-max) were recorded over time. The above experiment was performed with samples calcined at 100, 300, 600, and 900 °C. The photocatalytic reaction with NiO-SnO_2_ NCs calcined at 100 °C was repeated several times with different catalyst doses, pHs, and initial MO concentrations to see their effect on the photodegradation process [15,22,47].

## 4. Conclusions

NiO-SnO_2_ NCs were successfully synthesized via a biological route using a *F. elastica* leaf extract. XRD revealed that the prepared nanocomposite is composed of a rhombohedral NiO and tetragonal SnO_2_ nanostructure. The diffraction peaks become intense and sharp, suggesting that the degree of crystallinity increases with an increased calcination temperature. The crystallite size was gradually increased with an increased calcination temperature. The energy band gap decreased, and the wavelength increased with an increased calcination temperature. NiO-SnO_2_ NCs can degrade MO. The photocatalytic performance was found to decrease with an increased calcination temperature. This was due to an increase in the crystallite size of the NiO-SnO_2_ nanocomposites. The optimal conditions for the efficient photocatalytic activity of the biosynthesized NiO-SnO_2_ NC-mediated degradation of MO are 100 °C, catalyst dose = 20 mg, initial MO concentration = 50 ppm, and pH = 6.

This auspicious performance of the NiO-SnO_2_ NCs may open a new avenue for developing semiconducting p–n junction catalysts as promising structures for removing undesirable organic pollutants from the environment.

## Data Availability

Data sharing is available upon request from the corresponding author.

## Supplementary Materials

The following supporting information can be downloaded at: https://www.mdpi.com/article/10.3390/molecules27238420/s1, Figure S1: Collection of plant material samples (leaves of *F. elastica*). The area from where plant material samples were collected are shown in purple. Obtained from https://mapcarta.com/N2925426881, accessed on 20 September 2022.

## Sample Availability

Samples of the compounds are available from the authors.
