# Supplementary material for: Green Synthesis of NiO-SnO2 Nanocomposite and Effect of Calcination Temperature on Its Physicochemical Properties: Impact on the Photocatalytic Degradation of Methyl Orange
Source: Molecules. 2022 Dec 1;27(23):8420. doi: 10.3390/molecules27238420 (PMC9737821; doi:10.3390/molecules27238420)
Supplement: Supplementary file 1 [file molecules-27-08420-s001.zip › molecules-1955575-supplementary.pdf]

Supplementary Materials

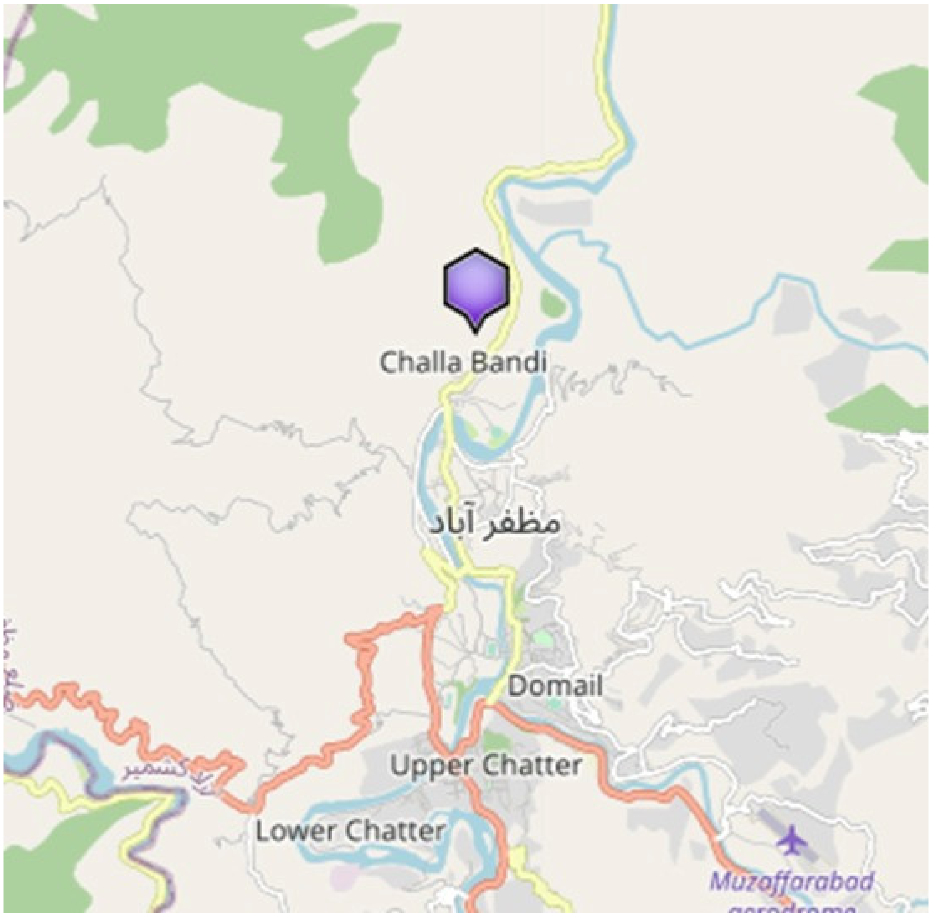

Figure S1. Collection of plant material samples (leaves of *F. elastica*). The area from where plant material samples were collected are shown in purple. Obtained from https://mapcarta.com/N2925426881, accessed on September 20, 2022.
